# The Control of Tuberculosis

**Published:** 1899-01-28

**Authors:** 


### The Hospital

A Journal of

The Medical Sciences and Hospital Administration.

Vol. XXV.—No. 644.] Edited by Sir Henry Burdett, K.C.B., and Solomon C. Smith, M.D., M.R.C.P. [Jan. 28, 1899.

### THE CONTROL OF TUBERCULOSIS.

Few things are likely to prove more perplexing to the historian of the future than the way in which England, after having for a long period occupied the van of sanitary progress, suddenly permitted herself to be displaced from that position by nations in most respects her inferiors, but whose rulers were wise enough to be guided, in purely scientific matters, by the light of scientific knowledge, rather than by the dictates of fancied political expediency. We say "fancied" advisedly, because the one lesson which politicians seem incapable of learning is that the quality most appreciated by Englishmen, in persons occupying positions of responsibility, is a courage adequate to the unflinching discharge of the duties which such positions impose. There can be no doubt that the Government of Lord Salisbury has lost more ground in the estimation of the great majority of the public by its conduct with reference to vaccination than it could have lost by any amount of weakness displayed in a more remote theatre of action, or where the conditions were less simple or less easily understood. Even the opponents of vaccination would have been compelled, if the Government had held their ground, to respect them as men with the courage of their convictions; whereas the only light in which they can now be looked upon is as persons who can be scared from any entrenchment, however impregnable, by any ragged regiment which presents even the slightest resemblance to an organised force. It is in perfect harmony with such a state of things that we should be told not to expect any assistance from legislation in diminishing the prevalence of tuberculosis. Legislation is demanded, alike by the facts of science and in the interests of humanity, but it might be resented by certain classes of voters, and to this consideration every other must give way.

In other countries, notably in Denmark, and in that sturdy outgrowth from Great Britain, the colony of New Zealand, matters are being very differently managed. Coincidentally with the establishment of the fact that an important share in the dissemination of tuberculosis is taken by infected milk and milk products, these are being protected against the invasion of the bacillus, or freed from its presence, by methods which are both enforced by law and demanded by public opinion. In Denmark, the use of the tuberculin test is an essential part of dairy management; the cattle shown by it to be infected are strictly separated from the healthy, and the sheds and byres are so arranged for all as to give ample breathing space in front of the muzzles of the animals, so that they may not be compelled, as in many English sheds, to breathe the same air over and over again, as it is reflected from a wall almost in contact with their nostrils. The fresh milk, too, is heated by proper mechanical arrangements, such as steam pipes, to a degree sufficient to kill any bacilli which it may contain, and is then artificially cooled and set aside, with the important results that while it throws up cream much more rapidly, it keeps much longer than any milk which has not been so treated. The heating, of course, not only destroys the bacillus of tubercle, but other forms which by their presence would occasion or promote decomposition. The importance of some system of this kind was completely established by the last research in which the lamented Professor Kanthack was engaged before his death, and in which he showed to how large an extent the milk and cream supplied to colleges in Cambridge were loaded with tubercle bacilli. The milk of nine dairies out of sixteen produced tuberculosis by inoculation into guinea pigs, and this equally whether the inoculated material was taken from the creamy layer or from the sediment. The *Lancet* for the 14th instant, in which an account of the Cambridge experiments is given by Professor Kanthack's coadjutor, Dr. Sladen, contains also a paragraph describing the detection of the tubercle bacillus in its most virulent form in a large number of samples of butter purchased at the shop of the principal butter merchant in Berlin. Attention has quite lately been called to the excess of English imports over exports as a condition striking at the root of our commercial prosperity, and it is well known that among these imports dairy produce holds a very considerable place. The British tradesman is already recognising existing conditions by selling home-made butter as "Danish," and the more foreigners are prepared to furnish us with safe articles the less we shall be likely to support the home producer, so long as he revels in his liberty to imperil the lives of his customers, and sl o n g as the pusillanimity of politicians refuses to take this liberty away.

